# β adrenergic receptor/cAMP/PKA signaling contributes to the intracellular Ca^2+^ release by tentacle extract from the jellyfish *Cyanea capillata*

**DOI:** 10.1186/s40360-017-0167-0

**Published:** 2017-07-25

**Authors:** Qianqian Wang, Hui Zhang, Bo Wang, Chao Wang, Liang Xiao, Liming Zhang

**Affiliations:** Department of Marine Biotechnology, Faculty of Naval Medicine, Second Military Medical University, Shanghai, 200433 China

**Keywords:** *Cyanea capillata*, Tentacle extract, β adrenergic receptor, Heart dysfunction, Propranolol

## Abstract

**Background:**

Intracellular Ca^2+^ overload induced by extracellular Ca^2+^ entry has previously been confirmed to be an important mechanism for the cardiotoxicity as well as the acute heart dysfunction induced by jellyfish venom, while the underlying mechanism remains to be elucidated.

**Methods:**

Under extracellular Ca^2+^-free or Ca^2+^-containing conditions, the Ca^2+^ fluorescence in isolated adult mouse cardiomyocytes pre-incubated with tentacle extract (TE) from the jellyfish *Cyanea capillata* and β blockers was scanned by laser scanning confocal microscope. Then, the cyclic adenosine monophosphate (cAMP) concentration and protein kinase A (PKA) activity in primary neonatal rat ventricular cardiomyocytes were determined by ELISA assay. Furthermore, the effect of propranolol against the cardiotoxicity of TE was evaluated in Langendorff-perfused rat hearts and intact rats.

**Results:**

The increase of intracellular Ca^2+^ fluorescence signal by TE was significantly attenuated and delayed when the extracellular Ca^2+^ was removed. The β adrenergic blockers, including propranolol, atenolol and esmolol, partially inhibited the increase of intracellular Ca^2+^ in the presence of 1.8 mM extracellular Ca^2+^ and completely abolished the Ca^2+^ increase under an extracellular Ca^2+^-free condition. Both cAMP concentration and PKA activity were stimulated by TE, and were inhibited by the β adrenergic blockers. Cardiomyocyte toxicity of TE was antagonized by β adrenergic blockers and the PKA inhibitor H89. Finally, the acute heart dysfuction by TE was antagonized by propranolol in Langendorff-perfused rat hearts and intact rats.

**Conclusions:**

Our findings indicate that β adrenergic receptor/cAMP/PKA signaling contributes to the intracellular Ca^2+^ overload through intracellular Ca^2+^ release by TE from the jellyfish *C. capillata*.

## Background

Jellyfish is one of the main groups in the marine biological species and ecosystem diversity. In recent years, the jellyfish explosion is no longer an unfamiliar topic, but became another marine ecological disaster after the red tides. Jellyfish outbreaks cause harm to fishery resources and marine ecosystem [1, 2], and negatively affect human beings who live near coast. What’s more, jellyfish sting is the most common injury by marine organisms, with an estimated 150 million envenomation patients annually [3], and the victims may suffer from a severe pain, itch, swelling, inflammation, dyspnea, arrhythmias, cardiac failure, pulmonary edema or even death [4–6].

Jellyfish envenomation has been attributed to the release of toxins from nematocysts in tentacles, and the injuries provoked by jellyfish are characterized by cytotoxicity [7–10], cardiotoxicity [11, 12], neurotoxicity [13], myotoxicity [14] and other toxicities. However, the most potent pharmacological effect exhibited by jellyfish venoms is on the cardiovascular system, and an acute heart failure caused by the cardiotoxicity of jellyfish venom is the major cause of fatal jellyfish envenomation [15]. Attempts to purify toxins from jellyfish venoms have been largely unsuccessful, and only approximately several hemolytic proteins have been purified and just a few of them have been characterized [16–20]. There was little information about the cardiotoxic component of jellyfish venoms, due to its extreme instability and the difficulties of toxin purification. Some studies have been carried out about jellyfish cardiovascular toxicity, describing the chemical and biological properties of the toxic components and their intervention by several different reagents, and the results have exhibited that a sudden influx of Ca^2+^ into heart muscle cells is an important reason for cardiovascular collapse [21, 22].

We have previously confirmed that extracellular Ca^2+^ entry plays a pivotal role in acute heart failure by tentacle extract (TE) from the jellyfish *Cyanea capillata*. Inhibition of extracellular Ca^2+^ influx is a promising antagonistic alternative for heart injuries by jellyfish venom [23]. In this study, we are aiming to update the current information on the mechanism of cardiotoxicity of TE from the jellyfish *C. capillata*. Our study revealed that β adrenergic receptor (βAR)/ cyclic adenosine monophosphate (cAMP)/ protein kinase A (PKA) signaling contributes to the intracellular Ca^2+^ release by TE from the jellyfish *C. capillata*. β blockades appear to exert anti-adrenergic effects against the cardiotoxicity of TE from the respects of fluorescent Ca^2+^ scanning, cardiomyocyte toxicity, isolated Langendorff-perfused rat hearts and intact rats.

## Methods

### Preparation of TE from the jellyfish *C. capillata*

Specimens of *C. capillata* were collected in June 2015 in the Sanmen Bay, East China Sea, and identified by Professor Huixin Hong from the Fisheries College of Jimei University (Xiamen, China). The removed tentacles were preserved in plastic bags on dryice and immediately shipped to Shanghai, where the samples were frozen at −70 °C untill use. The TE was prepared following the method as described in previous reports [24]. Briefly, frozen tentacles were thawed at 4 °C and immersed in seawater (prepared in the laboratory by solving 28 g of NaCl, 5 g of MgCl_2_·6H_2_O, 0.8 g of KCl and 1.033 g of CaCl_2_ in 1, 000 ml water) at a mass/volume ratio of 1:1 to allow autolysis of the tissues for 4 days. The mixture was stirred for 30 min twice daily. The autolyzed mixture was filtered by a 100 mesh cell strainer thrice and the filtrate was centrifuged at 10,000×*g* for 15 min thrice. The resultant supernatant was the TE. All the procedures were performed at 4 °C or in an ice bath. TE was centrifuged at 10,000×*g* for 15 min to remove the sediments, followed by dialysis against phosphate buffer saline (PBS) (0.01 mol/L, pH 7.4) for 8 h before use. Protein concentration was determined using the method of Bradford [25], with fetal bovine serum albumin as a standard.

### Adult mouse cardiomyocyte isolation

Single cardiomyocytes were obtained from adult male Kunming mice (22–25 g) using an enzymatic dissociation technique [26]. The hearts were excised from heparinized and deeply anaesthetized mice, cannulated and mounted on a Langendorff apparatus. After a digest perfusion for 8–10 min with perfusion buffer (mM: 10 HEPES, 0.6 Na_2_HPO_4_, 113 NaCl, 4.7 KCl, 12 NaHCO_3_, 0.6 KH_2_PO_4_, 1.2 MgSO_4_∙7H_2_O, 10 KHCO_3_, 30 Taurine, 10 2,3-Butanedine monoxime, 5.5 Glucose, pH 7.46) containing 773 U/ml collagenase type II (Worthington, USA), the ventricular tissue was cut into small pieces and gently stirred in stopping buffer containing perfusion buffer, 10% fetal bovine serum and 12.5 μM CaCl_2_ for 10–15 min, then transfered the upper cell suspension to a 25 ml beaker. After reintroduction the Ca^2+^ to a final concentration of 1 mM, the collected cells were kept at room temperature until experimental use.

### Measurement of intracellular Ca^2+^ by laser scanning confocal microscope (LSCM)

The intracellular Ca^2+^ imaging in cardiomyocyte was performed using an Olympus FV1000 confocal microscope (Olympus, Japan). The adult mouse cardiomyocytes were loaded with Fluo-4 AM (25 μM, Invitrogen) for 20 min to indicate the intracellular Ca^2+^. Under extracellular Ca^2+^-free or Ca^2+^-containing conditions, the cardiomyocytes after TE treatment (60 μg/ml) were ratiometrically scanned with an excitation wavelength at 488 nm and emission wavelength longer than 505 nm for 10 min with a 10-s interval. To confirm the participation of β adrenergic signaling in TE-induced intracellular Ca^2+^ overload, the cardiomyocytes were pre-incubated for 5 min with three different types of β blockers propranolol, atenolol and esmolol, and the PKA inhibitor H89, respectively.

### Primary neonatal rat cardiomyocyte incubation

Primary neonatal rat ventricular cardiomyocytes (NRVMs) were isolated from 1 to 2-day-old Sprague–Dawley (SD) rats by type II collagenase digestion. Briefly, the ventricles were excised, cut into small pieces, and incubated for 20 min thrice in D-Hanks’ balanced salt solution (g/L: 8 NaCl, 0.4 KCl, 1 Glucose, 0.06 KH_2_PO_4_, 0.0475 Na_2_HPO_4_) containing 0.125% collagenase type II. The detached cells were collected and gradient centrifuged for 26 min at 2800×*g* with a separation medium 60% percoll (at the bottom) and 40% percoll (at the top) in D-Hanks’ balanced salt solution. Then the cell suspension was collected and filled with the Dulbecco’s modified Eagle’s medium (DMEM) to centrifuge at 1500×*g* for 10 min. The isolated cardiomyocytes were plated at a density of 5 × 10^5^ cells/ml in DMEM supplemented with 20% fetal bovine serum in the presence of 0.1 mM 5-bromo-2-deoxyuridine (BrdU). Cell cultures were maintained for 48 h at 37 °C with 5% CO_2_ prior to being used in the experiments.

### cAMP assay

The intracellular cAMP concentration was determined by an ELISA kit (Ray Biotech, Inc., USA) in different treatments. The NRVMs were planted in a 24-well plate with the cell density 5 × 10^5^ cells/ml. After the intervention of β adrenergic receptor blockers (propranolol, atenolol and esmolol, respectively) for 5 min, the TE (60 μg/ml) was added and the myocardial cells were incubated for 45 min. After treatment, the cells were washed gently with ice-cold PBS, and lysed with RIPA buffer (Beyotime, China). The supernatant was obtained by a centrifugation of 10,000×*g* for 10 min at 4 °C. The concentration of cAMP was determined according to the manual of cAMP ELISA kit. Values for the changes of cAMP concentration were calculated as A/A0, where A was the cAMP concentration of intervention group and A0 was that of control group without any treatment.

### Determination of PKA activity

Primary neonatal rat cardiomyocytes were planted in 24-well plates at a density of 5 × 10^5^ cells/well, the cell lysate was obtained as described above. After the β blocker treatment for 5 min at 37 °C in a humidified incubator in HEPES containing TE (60 μg/ml), the PKA activity of cardiomyocytes was measured using an ELISA kit (Enzo Life Sciences International, Inc., USA).

### Cardiomyocyte toxicity of TE and its intervention

NRVMs were treated with TE from the jellyfish *C. capillata* for 24 h at the dose of 60 μg/ml. Cardiomyocyte toxicity of TE was evaluated with the Cell Counting Kit-8 (CCK-8, Tokyo, Japan). Three β blockers, including propranolol, atenolol and esmolol, and PKA inhibitor H89 were pre-administrated 30 min before the TE administration. After each treatment, 10 μl of CCK-8 solution was added and the cells were continuously incubated at 37 °C for 2 h. The absorbance of each well was measured with a spectrophotometric microplate reader at 450 nm by the following equation: viability% = [(AS-AB)/(AC-AB)] × 100%, where AS is the absorbance of the samples, AC is the absorbance of the PBS, and AB is the absorbance of the background.

### Isolated Langendorff-perfused rat hearts

Male SD rats (280–320 g) were anaesthetized with a mixture of urethane (1.2 g/kg i.p.) and heparin (400 IU/kg i.p.). The heart was rapidly excised and placed into an ice-cold Krebs solution. Then the heart was mounted onto a Langendorff apparatus and retrogradely perfused using a peristaltic pump (MPA-2000, Alcott Biotech, Shanghai, China) with warm Krebs solution (37 °C, pH 7.40). The Krebs solution, equilibrated with a mixture of 95% O_2_ and 5% CO_2_, was adjusted to keep a constant flow rate of 12–16 ml/min, thus maintaining an initial perfusion pressure of 60–80 mmHg. A fluid-filled balloon was introduced into the left ventricle through a polyethylene cannula. The initial balloon volume was set to generate the Lvedp of 2–8 mmHg. After a 20-min stabilization period, the isolated perfused heart was pre-treated with 1 μmol/L propranolol followed by the TE administered in bolus (180 μg). Heart indexes, including heart rate (HR), left ventricular developed pressure (LVDP), left ventricular end-diastolic pressure (LVEDP) and maximal ascending and descending derivative of left ventricle (± dP/dt_max_), were recorded/calculated by the MPA-2000 bio-signal analysis system.

### In vivo preparation

Effects of the venom and propranolol on blood pressure and heart rate were evaluated in male SD rats (220–250 g, provided by the Laboratory Animal Center of the Second Military Medical University, Shanghai) anaesthetized with urethane (1.2 g/kg i.p.). Heparinized polyethylene catheters were implanted into the left femoral artery and external jugular vein for mean arterial pressure (MAP) recording and venom injection, respectively. During all experiments, animals breathed spontaneously. 0.75 mg/kg propranolol was pre-administrated, followed by 0.5 mg/kg TE through the external jugular vein in intact rats. The mean blood pressure (BP) was recorded through a blood pressure transducer (Alcott Biotech, Shanghai, China) and signals were processed using the MPA-2000.

### Statistics

All the quantitative data were expressed as mean ± SD. The graphs were built using GraphPad Prism 5 for each experiment. Analysis of variance (ANOVA) was used to analyze the effects of TE administration and the invention either by β blockers or H89. A statistical significance was indicated by *P* < 0.05.

## Results

### Intracellular Ca^2+^-overload induced by TE

**Fig. 1:**
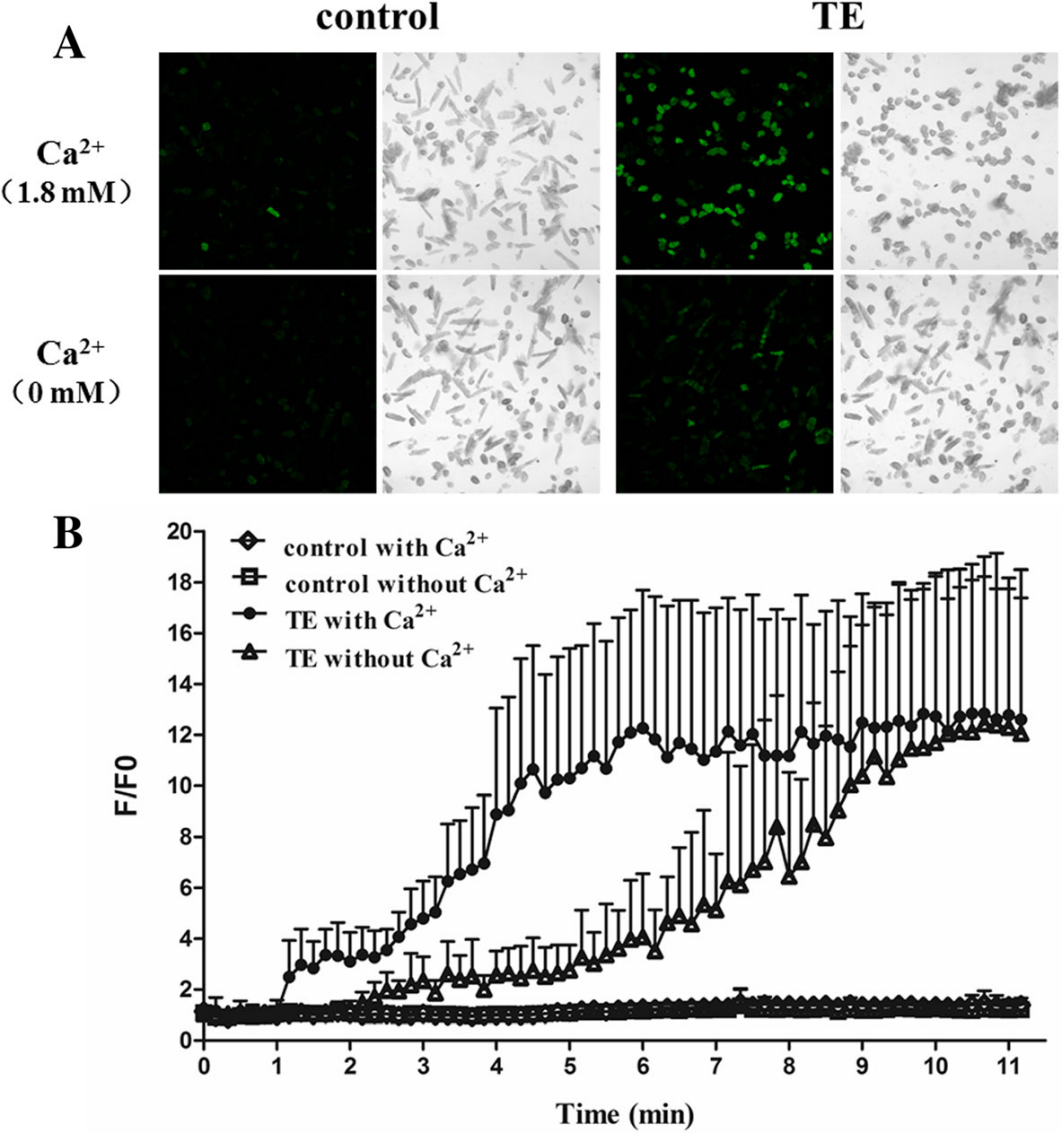
Effect of tentacle extract (TE) from jellyfish *Cyanea capillata* on intracellular Ca^2+^ concentration in adult mouse cardiomyocytes. Intracellular Ca^2+^ concentration was recorded using the Ca^2+^ indicator Fluo-4 AM and frame scanning mode by laser scanning confocal microscope. **a.** The representative fluorescent Ca^2+^ scanning images of cardiomyocytes affected by 60 μg/ml TE within 10 min under 1.8 mM Ca^2+^ extracellular solution and Ca^2+^-free condition. **b.** The traces of F/F0 values after TE administration under 1.8 mM extracellular Ca^2+^ or Ca^2+^ free condition. Data are shown by mean ± SD (*n* = 6)

We have previously displayed that extracellular Ca^2+^ entry plays a pivotal role in acute heart failure by TE from jellyfish *C. capillata*. In this study, fluorescent Ca^2+^ indicator Fluo-4 AM and frame scanning of confocal microscopy were used to firstly record the intracellular Ca^2+^ concentration affected by TE in adult mouse cardiomyocytes. Congruously, the signal of Ca^2+^ fluorescence started to rise at less than 1 min, and got to the maximum with a very bright vision at around 5 min after 60 μg/ml TE administration. The F/F0 got to and stayed at an approximate maximum value of 12 until cell death that normally occurred 15 min later. To exclude the influence of extracellular Ca^2+^ entry through either Ca^2+^ pore or Na^+^-Ca^2+^ exchanger, we repeated the experiments in a bath solution without Ca^2+^. Intracellular Ca^2+^ still increased after 60 μg/ml TE administration, just in a much slower and weaker manner. The time-to-maximum was at about 10 min after TE administration, with a peak F/F0 value very close to that induced by TE under 1.8 mM extracellular Ca^2+^ condition. In addition, the cardiomyocytes under Ca^2+^ free condition went to die at least 30 min later, or even without morphological changes and cell death (Fig. 1). These results showed the importance of extracellular Ca^2+^ entry in the process of intracellular Ca^2+^ overload induced by TE, on the other hand, the phenomenon of TE-induced intracellular Ca^2+^ release under Ca^2+^ free condition drew our attention.

### TE-induced intracellular Ca^2+^-overload hindered by β-blockade

**Fig. 2:**
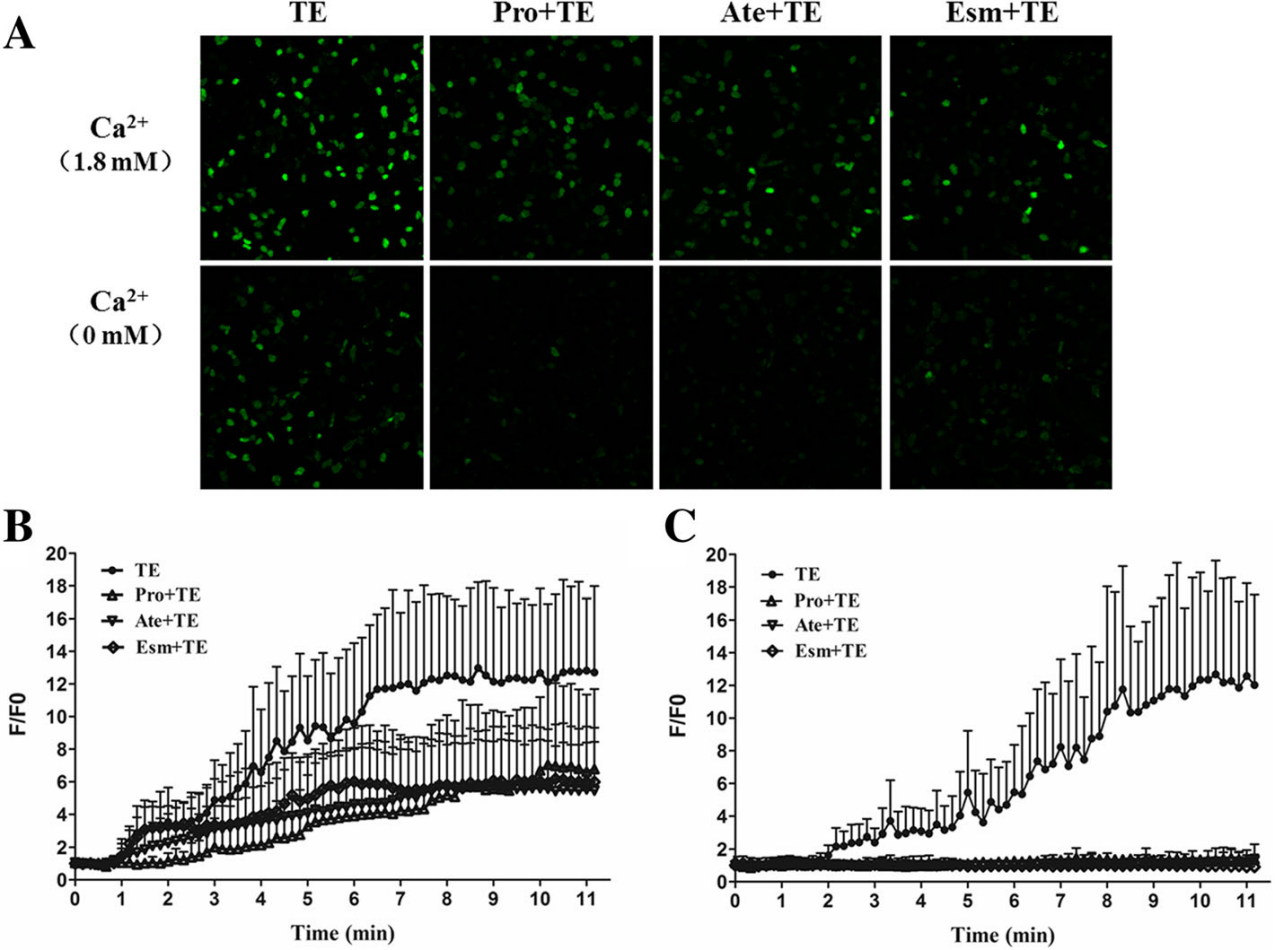
Effect of β blockers on TE-induced Ca^2+^ increase in isolated adult mouse cardiomyocytes. β-blockers propranolol, atenolol and esmolol were preincubated for 5 min with the cardiomyocytes followed by 60 μg/ml TE administration with or without 1.8 mM extracellular Ca^2+^. **a.** The representative fluorescent Ca^2+^ scanning images of cardiomyocytes affected by the β blockers on TE-induced intracellular Ca^2+^ overload under 1.8 mM Ca^2+^ extracellular solution and Ca^2+^-free condition. **b** The traces of F/F0 values affected by the β blockers on TE-induced intracellular Ca^2+^ overload under 1.8 mM extracellular Ca^2+^ condition (*n* = 6). **c** The traces of F/F0 values affected by the β blockers on TE-induced intracellular Ca^2+^ overload under extracellular Ca^2+^ free condition. Data are shown by mean ± SD (*n* = 6)

During the process of cardiac excitation-contraction (E-C) coupling, sympathetic excitation through β adrenergic signaling stimulates both Ca^2+^ release of cardiac ryanodine receptors (RyR2s) and Ca^2+^ uptake through sarcoplasmic reticulum Ca^2+^ pump, therefore enhancing both contraction and relaxation with fear, stress, exercise or excitation. Here β blockers, including propranolol, atenolol and esmolol, were used to check if TE has a pseudo adrenergic effect that stimulates the intracellular Ca^2+^ release in isolated adult mouse cardiomyocytes. Under the 1.8 mM extracellular Ca^2+^ condition, all the β blockers showed a partial inhibitory effect on TE-induced intracellular Ca^2+^ overload. In the β blocker groups, the elevation of intracellular Ca^2+^ was much slower with an obviously delayed time-to-peak value, and the maximal F/F0 of fluorescent Ca^2+^ signals was around 5, much smaller than that of the control. To our great surprise, all the three blockers almost abolished the elevation of intracellular Ca^2+^: we did not see any increase of intracellular Ca^2+^ within 10 min after TE administration (Fig. 2). Therefore, the results that β-blockade hindered the elevation of intracellular Ca^2+^ caused by TE indicated that β adrenergic singling might mediate the TE-induced intracellular Ca^2+^ release, contributing to the acute heart injuries after jellyfish envenomation.

### cAMP

**Fig. 3:**
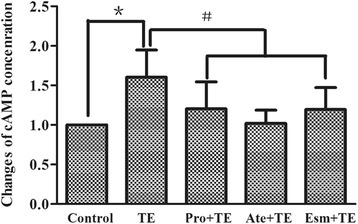
Effect of TE on cAMP concentration and its intervention by β blockers. The cAMP concentration was tested by ELISA in primary neonatal rat cardiomyocytes. The cells were pretreated for 5 min with β-blockers (propranolol, atenolol and esmolol, respectively) before TE administration (60 μg/ml, 45 min). The data are expressed as mean ± SD (*n* = 6). ^*^ *P* < 0.05 vs. the control. ^#^ *P* < 0.05 vs. TE

The concentration of the second messenger cAMP that is associated with the β adrenergic stimulation was tested by ELISA in primary neonatal rat cardiomyocytes. TE significantly increased the concentration of cAMP which reached 1.5 times of that in the control. All the β blockers, including propranolol, atenolol and esmolol, prevented the cAMP increase, and no difference was seen among the three groups (Fig. 3), further supporting that TE was able to activate the β adrenergic signaling.

### PKA activity

**Fig. 4:**
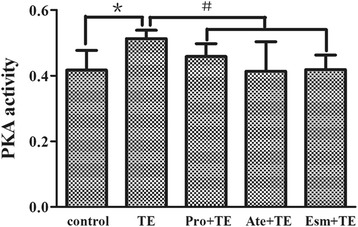
Effect of TE on PKA activity and its intervention by β blockers. The PKA activity was tested by ELISA in primary neonatal rat cardiomyocytes. The cells were pretreated for 5 min with β-blockers (propranolol, atenolol and esmolol, respectively) before TE administration (60 μg/ml, 45 min). Data are expressed as mean ± SD. ^*^ *P* < 0.05 vs. the control. ^#^ *P* < 0.05 vs. TE

**Fig. 5:**
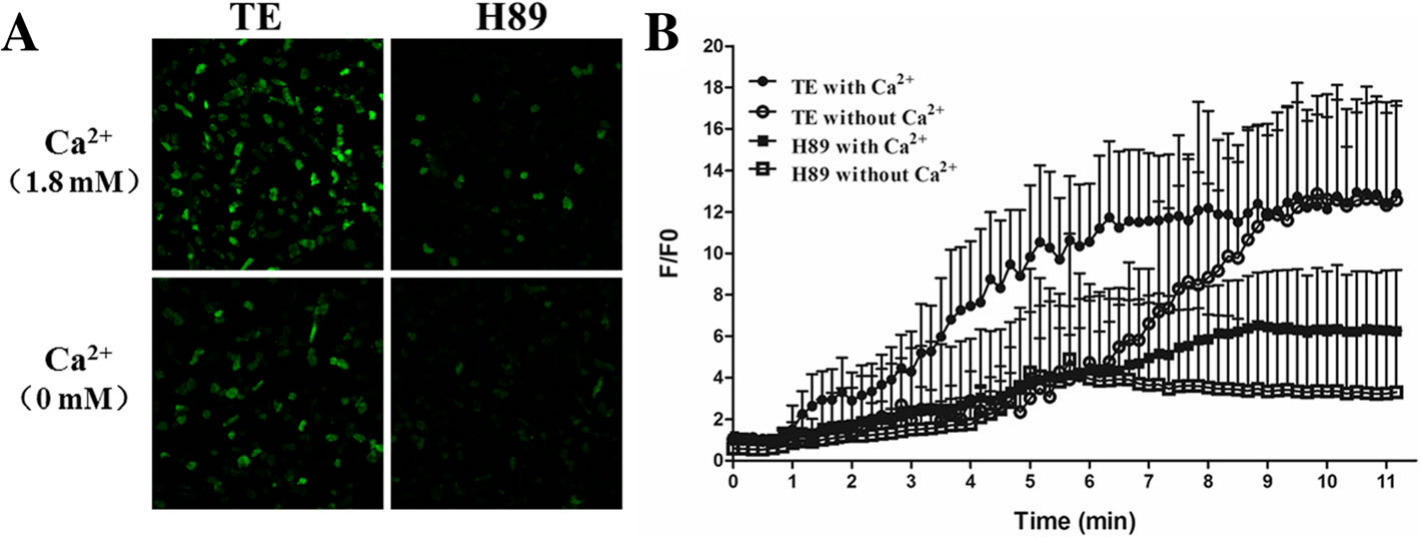
Effect of H89 on TE-induced intracellular Ca^2+^ increase in isolated mouse cardiomyocytes. The PKA inhibitor H89 was preincubated for 5 min with the cardiomyocytes followed by 60 μg/ml TE administration with or without 1.8 mM extracellular Ca^2+^. **a.** The representative fluorescent Ca^2+^ scanning images of cardiomyocytes affected by H89 on TE-induced intracellular Ca^2+^ increase. **b.** The traces of F/F0 values affected by H89 on TE-induced intracellular Ca^2+^ increase (*n* = 6). Data are shown by mean ± SD (*n* = 6)

The principal function of cAMP on Ca^2+^ homeostasis during sympathetic excitation is to activate PKA activity. Similar with cAMP concentration, PKA activity in primary neonatal rat cardiomyocytes increased after TE administration, which was also antagonized by the β blockers propranolol, atenolol and esmolol (Fig. 4). Meanwhile, the PKA inhibitor H89 that cutoff the relationship between β adrenergic receptor and Ca^2+^ release brought about an expected lower and delayed elevation of intracellular Ca^2+^ in isolated mouse cardiomyocytes (Fig. 5). Combing the above results, a frame of βAR/cAMP/PKA pathway that mediates the intracellular Ca^2+^ release by TE emerges.

### Antagonistic effects of βAR blockers on TE-induced cardiomyocyte toxicity

**Fig. 6:**
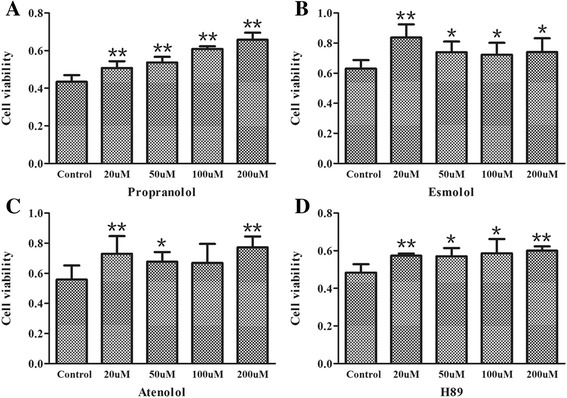
Cardiomyocyte toxicity induced by TE and its intervention by β blockers and H89. Primary neonatal rat cardiomyocytes were preincubated for 30 min with β-blockers propranolol (**a**), atenolol (**b**) and esmolol (**c**), and PKA inhibitor H89 (**d**), respectively, followed by 60 μg/ml TE administration, then the cell viability was evaluated by CCK8 assay. Data are expressed as mean ± SD (*n* = 8). ^*^ *P* < 0.05 vs. the control, ^**^ *P* < 0.01 vs. the control

By CCK8 assay, we further analyzed the antagonistic effects of the β blockers and PKA inhibitor H89 on cardiomyocyte toxicity of TE in primary neonatal rat cardiomyocytes that was observed by us previously. All the reagents displayed a significant inhibition on the cardiomyocyte toxicity of TE (Fig. 6), further supporting that the intracellular Ca^2+^ release was mediated through βAR/cAMP/PKA signaling.

### In vitro effect of βAR blocker on TE-induced acute heart dysfunction

**Fig. 7:**
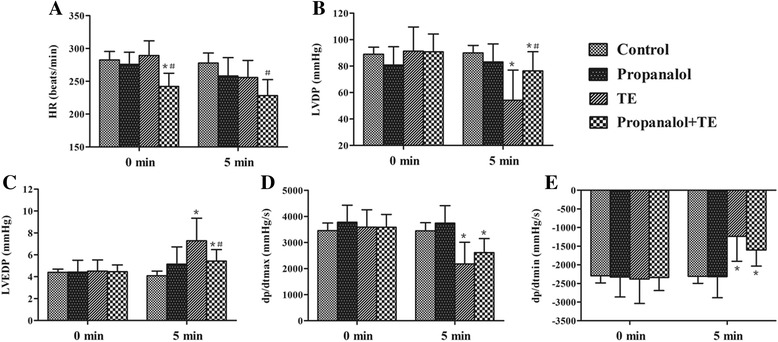
In vitro effects of propranolol on the TE-induced acute heart dysfunction. Propranolol at 1 μmol/L was pre-administrated followed by a dose of 180 μg TE in Langendorff-perfused rat hearts. Parameters including HR (**a**), LVDP (**b**), LVEDP (**c**), dp/dt_max_ (**d**) and -dp/dt_min_ (**e**) were recorded. Data are expressed as mean ± SD (*n* = 8), ^*^ *P* < 0.05 vs. the control, ^#^ *P* < 0.05 vs. TE

Propranolol was selected as a representative to examine the in vitro antagonistic effect of β blockers on the TE-induced acute heart dysfunction in isolated Langendorff-perfused rat hearts. Propranolol at 1 μmol/L was pre-administrated followed by TE at a dose of 180 μg through the external jugular vein. After TE administration, HR decreased from 293 ± 29 beats per minute (bpm) to 256 ± 26 bpm (Fig. 7a). LVDP declined significantly (Fig. 7b), LVEDP elevated obviously (Fig.7c), both +dP/dt_max_ and –dP/dt_min_ decreased accordingly (Fig.7d and e). All these indexes except HR were partially recovered in the propranolol-treated group (Fig. 7), indicating that propranolol could partially attenuate the TE-induced acute heart dysfunction in Langendorff-perfused rat hearts.

### In vivo effect of βAR blocker on the TE-induced decrease of blood pressure

**Fig. 8:**
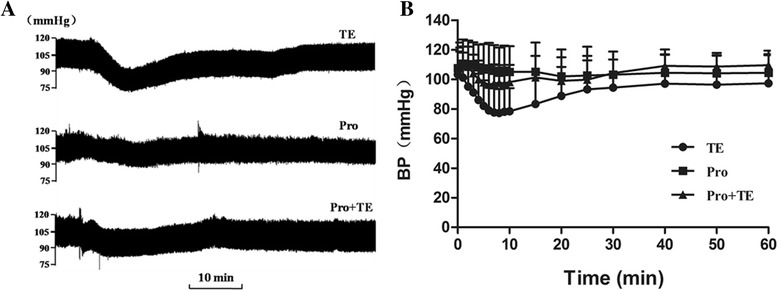
In vivo effects of propranolol on the TE-induced declination of blood pressure (BP). Propranolol at 0.75 mg/kg was pre-administrated, followed by 0.5 mg/kg TE through the external jugular vein in intact rats. The representative images (**a**) and parameters (**b**) of BP were displayed. Data are expressed as mean ± SD (*n* = 8)

Blood pressure was determined in order to evaluate the antagonistic effect of propranolol on the TE-induced acute heart dysfunction in intact rats. Pre-administration of 0.5 mg/kg propranolol greatly recovered the TE-induced decrease of blood pressure (Fig. 8), indicating that propranolol prevents against the acute heart dysfunction in vivo.

## Discussion

### Cardiotoxicity by jellyfish venom

The cardiotoxicity of jellyfish venom showed great variations. Patients with the famous Irukandij syndrome presented with a significant and ongoing pain but with no or minimal skin markings, tachycardia, hypertension followed by hypotension and pulmonary oedema, indicating a significant cardiac dysfunction and life-threatening induced by the jellyfish *Carukia barnesi* [27, 28]. Venom from Okinawan box-jellyfish (Habu-kurage), *Chiropsalmus quadrigatus*, produced an increase in contraction of isolated rat right atrial preparation in a concentration-dependent manner that was significantly inhibited by diltiazem [29]. Intravenous administration of the nematocyst venom from *Chiropsalmus quadrigatus* produced a dose-dependent hypertension and bradycardia immediately, followed by a significant depressive response of blood pressure and irreversible cardiac arrest in anaesthetized rats and rabbits, which were not affected by prazosin, atropine or BQ123 but attenuated by nicardipine [30, 31]. Intravenous injection of 10 μg/kg nematocyst venom produced a transient pressor response followed by cardiovascular collapse in anaesthetized rats whereas 100 μg/kg TE produced a more prolonged hypertensive effect without cardiovascular collapse that was attenuated by 50 μg/kg prazosin, indicating that a distinct difference of cardiovascular pharmacological effects between nematocyst venom and TE [32].

The manifestation of acute heart dysfunction reported by us from the TE of jellyfish *C. capillata* mainly displayed in three aspects, including HR decrease, systolic and diastolic dysfunction and imbalance between cardiac oxygen supply and demand. Both in vitro and in vivo investigation displayed that HR decreased dose-dependently that occurred immediately and partially recovered in 10 min after TE administration, indicating a blemish of the sinus node/conduction system [15, 33]. The significant decrease of BPs and LVPs implied a serious injury of left ventricle. Moreover, the serious decrease of EVR and elevation of ST-T segment in ECG indicated an imbalance between the cardiac oxygen supply and demand that might be attributed to the serious coronary spasm [34]. Thus, the acute heart dysfunction caused by TE from jellyfish *C. capillata* is related to the direct injuries of sinus node/conduction system, ventricular working cells, as well as myocardial ischemia and hypoxia by coronary spasm [23].

### Extracellular Ca^2+^ entry

The exact mechanism of the acute heart dysfunction is not clear, however, intracellular Ca^2+^ overload induced by extracellular Ca^2+^ entry has been congruously recognized as an important mechanism underlying the cardiotoxicity of jellyfish venom. As early as 1973, Calton and Burnett found the effect of two jellyfish toxins on calcium ion transport [35]. Since from 1983, the Ca^2+^ antagonists verapamil, diltiazem and nifedipine were sequentially reported to antagonize cardiotoxicity of the venom from *Chironex fleckeri* [36], Portuguese man-o’war (*Physalia physalis*) [37], *Carybdea rastonii* [38, 39] or *C. capillata* [33]. However, another study reported that the verapamil had no effect on Ca^2+^ influx whilst La^3+^, a non-specific channel and pore blocker, inhibited the Ca^2+^ influx, consistent with the presence of a pore-forming toxin existing in the venoms which was demonstrated by transmission electron microscopy in the case of *C. fleckeri* [21]. It was also reported that the venom from the jellyfish *C. fleckeri* caused extracellular Na^+^ entry and intracellular Ca^2+^ overload in cardiomyocytes that was not inhibited by blockers of Ca^2+^ or Na^+^ channels or by inhibitors of Na^+^/K^+^ ATPase or Na^+^/H^+^ exchange in the sarcoplasmic reticulum, but blocked by prior exposure to a solution which contained no Na^+^ and by Ni^2+^, indicating a possible mechanism of intracellular Ca^2+^ overload induced by jellyfish venom via the Na^+^/Ca^2+^ exchange [22].

TE from *C. capillata* led to an intracellular Ca^2+^ overload that was greatly weakened after removal of the Ca^2+^ in extracellular solution in this study, further supporting the importance of extracellular Ca^2+^ entry. Although the extracellular Ca^2+^ entry can be prevented by the L-type Ca^2+^ blockers, the mechanism of extracellular Ca^2+^ entry is now dominated by the existence of pore-forming complex in jellyfish venom that allows a non-specific translocation of Ca^2+^ across the cell membrane [10, 23]. So far, more than 10 hemolytic proteins have been identified as a novel protein family taxonomically restricted cnidarian toxins (42–46 kDa), from the jellyfish species *C. rastoni*, *C. fleckeri*, *Cyanea nozakii* Kishinouye and *Chironex yamaguchii* (as *C. quadrigatus*), respectively [7, 16, 18, 40, 41]. Bioinformatic analysis indicates that these novel proteins function as the non-selective cation pore-forming proteins from jellyfish venoms and thereby contributing to the extracellular Ca^2+^ entry. However, this is not the only situation, comparative functional analysis of the two pairs of structurally similar hemolytic proteins from *Chironex feckeri*, CfTX-1/2 and CfTX-A/B, showed that CfTX-1/2 caused profound effects on the cardiovascular system of anaesthetized rats, whereas CfTX-A/B elicited only minor cardiovascular effects but displayed a hemolytic activity at least 30 times greater than that of CfTX-1/2, indicating that the hemolytic proteins of jellyfish venoms have structural diversification and functional selection during evolution [41].

### βAR/cAMP/PKA signaling

The involvement of adrenergic system in the cardiovascular toxicity of jellyfish venom was firstly drew by the partial inhibition of the α-adrenergic antagonist phentolamine, the central antiadrenergic reagent trifluoperazine as well as the phosphodiesterase inhibitor papaverine on the aorta contraction induced by the venom of the jellyfish *C. rastonii* [39]. Further studies showed that the α1-adrenergic antagonist prazosin and β-adrenergic blocker propranolol attenuated the nematocyst venom-induced pressor response and tachycardia in anaesthetized rats [42, 43]. Similar results displayed that the pressor response induced by the venom of the jellyfish *Alatina nr mordens* was significantly inhibited by prazosin. However, these effects were not uniformed among different jellyfish species. The concentration dependent inotropic response in left atria induced by the crude venom of a newly described jellyfish *Malo maxima* causing Irukandji syndrome was significantly attenuated by propranolol but not by prazosin [44]. However, a prior administration of prazosin did not significantly attenuate the hypertensive response nor prevent the cardiovascular collapse induced by nematocyst venom of the jellyfish *Chiropsalmus* sp. [45]. Prazosin did not evidently affect the cardiovascular effects produced by nematocyst venom but significantly attenuated the pressor response produced by TE from *C. fleckeri* [32]. Another study on the venom from *C. fleckeri* displayed that both propranolol and prazosin have no effect on the cardiovascular toxicity of the nematocyst venom [46]. Therefore, previous studies from other laboratories have confirmed that the adrenergic system mainly affected the pressor but not the depressor period induced either in vivo or in vitro with different jellyfish venoms.

In this study, we are interested in the intracellular Ca^2+^ overload through intracellular Ca^2+^ release that was induced by TE from the jellyfish *C. capillata*. This phenomenon was hindered by β adrenergic blockers propranolol, atenolol and esmolol. cAMP concentration and PKA activity were both increased correspondingly, implying the involvement of β adrenergic signaling in cardiotoxicity of jellyfish venom. We also forwarded our studies in Langendorff-perfused rat hearts, which have got the partial antagonistic effects of propranolol on the heart dysfunction. The reason for the negative results of adrenergic blockers from previous studies can be explained by the mask of significant extracellular Ca^2+^ entry through pore-performing components. In intact rats, we used a gentle dose of TE from *C. capillata* that only led to a BP decrease of approximate 20 mmHg, and thereby allowing to display the role of β adrenergic signaling as well as the antagonistic effect of propranolol. This result is consistent with the change of intracellular Ca^2+^, where propranolol only partially inhibited the intracellular Ca^2+^ overload with normal extracellular 1.8 mM Ca^2+^ while completely abolished the intracellular Ca^2+^ increase when the extracellular Ca^2+^ was removed from the bath solution.

Collectively, we have firstly confirmed the involvement of the β adrenergic signaling in the intracellular Ca^2+^ overload through intracellular Ca^2+^ release as well as the cardiotoxicity of jellyfish venom from the respects of fluorescence Ca^2+^ scanning, cardiomyocyte toxicity and acute heart dysfunction in Langendorff-perfused rat hearts and intact rats.

## Abbreviations

± dP/dt_max_: maximal ascending and descending derivative of left ventricle
BP: blood pressure
cAMP: cyclic adenosine monophosphate
DMEM: Dulbecco’s modified Eagle’s medium
HR: heart rate
LSCM: laser scanning confocal microscope;
LVDP: left ventricular developed pressure
LVEDP: left ventricular end-diastolic pressure
MAP: mean arterial pressure
NRVMs: neonatal rat ventricular cardiomyocytes
PBS: phosphate buffer saline
PKA: protein kinase A
RyR2s: ryanodine receptors
TE: tentacle extract
βAR: β adrenergic receptor
